# Future Expectations and Internet Addiction Among Adolescents: The Roles of Intolerance of Uncertainty and Perceived Social Support

**DOI:** 10.3389/fpsyt.2021.727106

**Published:** 2021-08-26

**Authors:** Gang Du, Houchao Lyu

**Affiliations:** Faculty of Psychology, Time Psychology Research Center, Southwest University, Chongqing, China

**Keywords:** future expectations, intolerance of uncertainty, perceived social support, internet addiction, adolescents

## Abstract

Internet addiction is a common and challenging problem among adolescents. Previous studies have shown that future time orientation is an important protective factor against internet addiction. In this study, the mediating effect of intolerance of uncertainty and the moderating role of perceived social support were examined on the association between future expectations, regarded as the “prospective life course” perspective of future time orientation, and internet addiction among adolescents. A total of 1,006 Chinese adolescents (54% male and 46% female; Mage = 15.42 years, SD = 1.32) recruited from middle schools completed questionnaires. Results indicated that future expectations were significantly negatively associated with internet addiction, and the link was mediated by intolerance to uncertainty in adolescents. Further, the latent moderated structural equation showed that perceived social support moderated the association between future expectations and intolerance of uncertainty. The association was significant only for adolescents with a higher level of perceived social support. The findings of this study provide specific guidelines for how to prevent adolescent internet addiction.

## Introduction

Internet surfing has become one of the most popular leisure activities among adolescents. More than 174 million Chinese adolescents aged 10–19 years are internet users, accounting for 19.3% of cyber citizens in China (1). The internet helps to expand adolescents' social networks and relieve their emotional distress. It may also lead to addiction, an excessive or compulsive internet usage characterized by a loss of personal control (2), which results in poor mental health or other maladaptive behaviors (3). Previous studies have shown that present-oriented factors (e.g., impulsiveness, sensory seeking, and obsessive passion) can lead to internet addiction (4). However, there are few studies on future-oriented and motivational factors (e.g., expectations, goals, aspiration). If individuals have low motivational factors, they would indulge in the present and find it hard to refuse pleasure-seeking in daily life (5). Consequently, the current study focused on the relationship between future expectations and internet addiction. Further, it examined the mediating effect of intolerance of uncertainty and the moderating role of perceived social support to provide suggestions for the prevention of and interventions for adolescent internet addiction.

### Future Expectation and Internet Addiction

Thinking about the future is an essential part of human cognition (6). Future expectations are defined as beliefs or expectancies about the likelihood of a specific event occurring in the future (7–9). Future expectations are considered synonymous with the “prospective life course” perspective of future time orientation (10), which tends to be task-oriented (e.g., family, education, career) and has been shown to help regulate behavior and emotional well-being (11).

Individuals' cognitive representations of future events could have implications for present behaviors (12). Expectancy-value theory (13), a self-regulation theory in motivational psychology, posits that expectations are crucial. Further, it assumes that individuals who have positive expectations for a goal tend to adopt approach behaviors and continuously shorten the distance between the present and the goal to be achieved (14). In this process, expectations serve as powerful motivators of current decisions that push individuals away from immediate enjoyment. Specifically, expectations stimulate individuals to consider the future consequences of their current behavior to devote themselves less to immediate rewards (e.g., excessive internet usage). Previous studies have shown that future time perspective negatively predicted Internet and Facebook addiction (15, 16). Furthermore, a positive future time perspective was a significant predictor of internet gaming disorder symptoms, either immediately or 3 years later (17). A recent meta-analysis revealed that future time perspective was positively associated with health-related behaviors, as indicated by a negative association with substance use (18). Therefore, this study hypothesized that future expectations are negatively associated with internet addiction.

**Figure 1:**
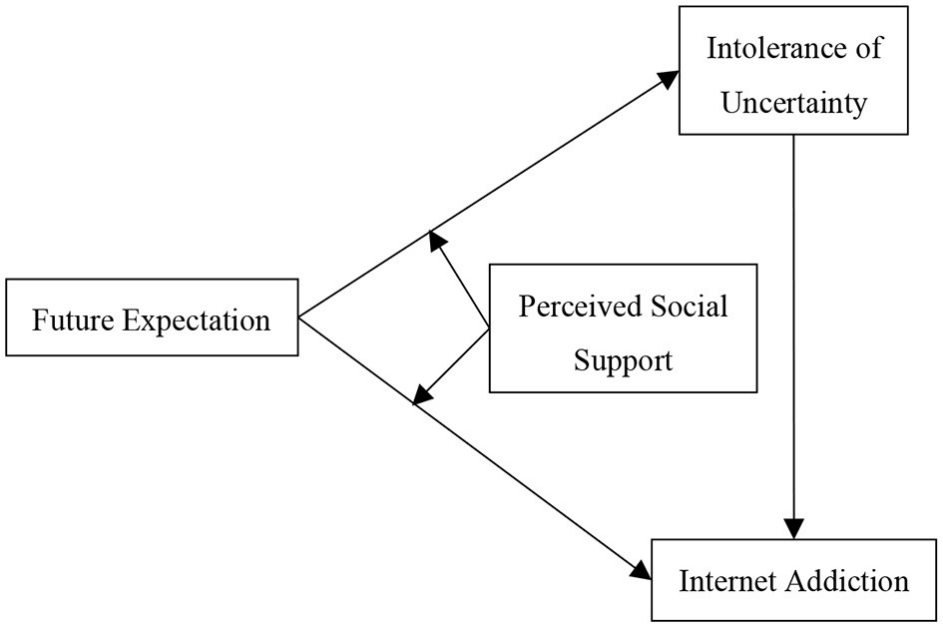
The proposed moderated mediation model.

Although studies have shown that future expectations may reduce or affect adolescents' internet addiction, few studies have examined the possible mechanisms that might underpin the association between future expectations and internet addiction. Potential mediators and moderators of the relationship between future expectations and internet addiction that may intensify or weaken the association are still unknown. The study expanded on previous research by proposing a moderated mediation model (see Figure 1), which may clarify the mediation and moderation processes underlying the association between future expectations and internet addiction.

### Intolerance of Uncertainty as a Mediator

Intolerance of uncertainty (IU) is defined as a cognitive bias that affects how a person perceives, interprets, and responds to uncertain situations at the cognitive, emotional, and behavioral levels (19). Future expectations may reduce one's level of IU. Although uncertainty can feel intolerable, humans' subjective initiatives can help them take control of their lives. They can adopt more proactive approaches (e.g., cognitive reappraisal and pre-planning) to cope with the anxiety of uncertainty. This means that people's positive advantages or expectations can play a vital role in some uncertain situations. van den Bos et al., (20) proposed an integrated model of coping with personal uncertainty, which points out that humans' ultimate goal is to pursue self-realization and actively seeking goals relieves personal uncertainty. Self-uncertainty can be regarded as temporary confusion or doubt in the process of following one's goals. McGregor et al., (21) showed that writing about how personal goals promote core values and identifications may relieve anxious uncertainty and prevent subsequent defensive reactions to personal uncertainty. Equally, as a prospective thought, future expectations should guide individuals toward established goals when faced with uncertainty. Consequently, it can be assumed that future expectations have an essential impact on IU.

Simultaneously, IU is a cognitive bias that could increase the possibility of excessive internet use. Due to the uncertainty and anxiety caused by rapid social changes and brutal social competition, many individuals lacking social competitiveness tend to seek temporary peace and detachment through the Internet. Luhmann et al., (22) observed that IU was associated with reward-based decision-making by utilizing a simple laboratory gambling task and found that higher IU was associated with a tendency to select the immediately available, but less valuable and less probable, rewards. With an increasing degree of IU, the probability of choosing immediate safety reward behavior would increase correspondingly (23). It was inferred that participants with higher IU are more inclined to engage in present hedonic behaviors and ignore these behaviors' long-term consequences.

Given the associations among future expectations, IU, and internet addiction described above, IU may act as a mediator of the relationship between future expectations and internet addiction. This means that future expectations may reduce the IU level (i.e., facilitating tolerance of uncertainty), thus alleviating adolescents' internet addiction.

### Perceived Social Support as a Moderator

Perceived social support (PSS) is defined as the perception of available resources/supportive social ties acquired *via* social interaction (24). It is usually regarded as a cumulative protective factor that considers the support of parents, teachers, and friends. According to the stress-buffering model (25), PSS can buffer individuals from the influence of stressful events or circumstances by providing appropriate coping responses. Individuals with high PSSs will experience greater feelings of being respected and understood in the face of uncertain situations. Previous studies have shown that one-on-one peer support intervention could facilitate uncertainty management and enhance psychosocial functioning in patients diagnosed with HIV (26). Conversely, individuals with low PSSs lack the necessary psychological resources to ineffectively deal with uncertain situations or face more maladaptive consequences (e.g., online game addiction, negative emotions).

Based on the above, it is proposed that PSS may serve as a moderator of the relationship between future expectations and internet addiction *via* IU. According to ecological systems theory, there is an interaction between individuals and the environment (27). Theoretically, it is feasible to analyze and verify the moderator role of PSS. Individual-context relations theory (28) also suggests that individual behaviors are formed and developed during the interaction between individuals and the environment surrounding them. Empirical studies have confirmed that the interaction between individual factors and PSS can affect individual mentalities. For example, PSS moderated the associations between fear of missing out and authentic adolescent self-presentation on social networking sites (29). The effects of cyber victimization on adolescent depression were moderated by PSS (30). These findings indicate that PSS may interact with individual factors (e.g., future expectations) to affect IU and internet addiction.

### The Present Study

The study examined a model of the process by which future expectations predict internet addiction among adolescents. In particular, (a) to test whether IU mediates the relationship between future expectations and internet addiction, and (b) to test whether PSS would moderate the indirect associations between future expectations and internet addiction through IU. Taken together, this integrated moderated mediation model can address questions about both mediation (i.e., how do future expectations relate to internet addiction?) and moderation (i.e., when or for whom is the relation most or least potent?) in a single model.

## Materials and Methods

### Participants and Procedures

A total of 1,006 middle school students (54% male and 46% female) participated in this survey. The participants were recruited from eight middle schools in China. The mean age was 15.42 years (SD = 1.32, range = 11–18 years). The results showed that 806 participants had mobile phones and internet access (accounting for 80.1 %), and 804 participants had computers and internet access at home (accounting for 79.9%). The participants' average online time was 1.84 h per day (SD = 2.07 h), and the average number of years was 5.84 years since they first got internet access (SD = 2.72 years).

The Ethics in Human Research Committee of the corresponding author's university approved all materials and procedures. Students were invited to participate in the survey anonymously in classrooms. Informed consent was obtained from each participant and school. Well-trained postgraduate psychology students collected the survey data on paper. The authenticity, independence, and integral nature of all answers were emphasized to the participants. Students were informed that they could terminate their responses anytime they wanted. Data collection was performed within 30 min to complete all the questions in the questionnaires.

### Measures

#### Future Expectations

Future expectations were assessed using the Future Expectations Scale for Adolescents (7), which has been translated into Chinese (31). The scale consists of 24 items and five dimensions, including work and education, marriage and family, social group, health, and children's futures. Sample items are “When I am an adult, I will achieve the level of education that I want” and “When I am an adult, I will have good health.” For each item, adolescents indicated the degree to which they agreed with the statement on a seven-point scale, ranging from 1 (I do not believe this at all) to 7 (I certainly believe this). The reliability coefficients for the total score (α = 0.92) and the subscales (α = 0.77-0.91) in the current study were satisfactory. Confirmatory factor analysis (CFA) showed that the scale fit the data quite well (χ^2^*/df* = 4.66, CFI = 0.93, TLI = 0.88, RMSEA = 0.07, 90% CI [0.07, 0.08], SRMR = 0.03).

#### Internet Addiction

Internet addiction was assessed using the Adolescent Internet Addiction Diagnosis Questionnaire (2), adapted for use in Chinese culture (30). The scale consists of 10 items (e.g., “I feel the need to use the internet with increasing amounts of time to achieve satisfaction”). Responses were made on a six-point scale, ranging from 1 (extremely disagree) to 6 (extremely agree). In this study, Cronbach's α coefficient (alpha) of the measure was 0.88. CFA showed that the scale fit the data quite well (χ^2^*/df* = 3.55, CFI = 0.99, TLI = 0.97, RMSEA = 0.051, 90% CI [0.038, 0.065], SRMR = 0.017).

#### Intolerance of Uncertainty

Intolerance of uncertainty was assessed with the Intolerance of Uncertainty Scale-Chinese version (32, 33). The scale consists of 12 items and three dimensions involving prospective anxiety, prospective behavior, and inhibitory behavior. Sample items are “Uncertainty keeps me from living a full life” and “Unforeseen events upset me greatly.” For each item, adolescents indicated the degree to which they agreed with the statement on a five-point scale, ranging from 1 (not my characteristic at all) to 5 (entirely my characteristic). The IUS dimensions (α = 0.65, 0.67, 0.78, respectively) and total score (α = 0.80) demonstrated good internal consistency in this study. CFA showed that the scale fit the data quite well (χ^2^/*df* = 5.01, CFI = 0.95, TLI = 0.90, RMSEA = 0.064, 90% CI [0.055, 0.074], SRMR =0.028).

#### Perceived Social Support

Perceived social support was assessed using the PSS Scale (34) and revised for Chinese participants (35). The scale consists of 12 items and three support domains: family, friend, and significant others. An example item is “I have friends with whom I can share my joys and sorrows.” For each of the 12 items, adolescents indicated the degree to which they agreed with the statement on a seven-point scale, ranging from 1 (extremely disagree) to 7 (extremely agree). Cronbach's α for the measure (α = 0.92) and its three facets (α = 0.85, 0.86, and 0.84, respectively) in the present sample were satisfactory. CFA showed that the scale fit the data quite well (χ^2^*/df* = 6.71, CFI = 0.97, TLI = 0.94, RMSEA = 0.077, 90% CI [0.068, 0.087], SRMR = 0.023).

### Data Analysis

The data were analyzed using Mplus 7.4 (36), which tested the hypothesized moderated mediation model with structural equation modeling (SEM). The bootstrap method based on 5,000 samples was used to obtain bias-corrected and accelerated 95% confidence intervals (CIs) (37). The 95% CI that does not include zero suggests a significant effect. The model fit was assessed using multiple fit indices, including ratio chi-square over degrees of freedom (χ^2^/*df*), comparative fit index (CFI), Tucker-Lewis index (TLI), root mean square error of approximation (RMSEA), and the standard root mean square residual (SRMR). The SEM study suggested a good model fit was defined by the following criteria: CFI ≥ 0.90, TLI ≥ 0.90, RMSEA ≤ 0.08, and SRMR ≤ 0.08 (38).

## Results

### Descriptive Statistics and Correlation Analysis

The skewness of the variables was between −0.37-0.50, and kurtosis was between −0.09 and 0.89, indicating that the variables conformed to the typical distribution hypothesis (39). Descriptive statistics and Pearson correlation analyses were conducted, and the results are shown in Table 1. The correlation analyses found that most future expectations were significantly correlated with dimensions of IU, PSS, and factors of internet addiction (see the measurement model). The dimensions of IU were significantly correlated with the dimensions of PSS and aspects of internet addiction. Further, the dimensions of PSS were significantly associated with the factors of internet addiction. Gender and age were correlated with future expectations and internet addiction, and were included as covariates in subsequent analyses.

**Table 1:** Descriptive statistics and correlations for all variables.

	**1**	**2**	**3**	**4**	**5**	**6**	**7**	**8**	**9**	**10**	**11**	**12**	**13**	**14**	**15**	**16**
1 Gender	-															
2 Age	−0.08*	-														
3 Work and education	−0.05	−0.01	-													
4 Marriage and family	−0.14***	−0.02	0.33***	-												
5 Children's future	0.04	0.02	0.34***	0.58***	-											
6 Social group	0.02	0.01	0.50***	0.23***	0.28***	-										
7 Health	−0.03	−0.02	0.50***	0.32***	0.39***	0.53***	-									
8 Prospective anxiety	−0.01	0.02	−0.19***	−0.11**	−0.06	−0.12***	−0.17***	-								
9 Prospective behavior	−0.02	0.04	−0.13***	−0.04	0.00	−0.09**	−0.10**	0.62***	-							
10 Inhibitory behavior	0.03	−0.04	−0.12***	−0.01	−0.05	−0.08*	−0.12***	0.51***	0.48***	-						
11 Family support	0.00	0.02	0.31***	0.16***	0.21***	0.25***	0.31***	−0.20***	−0.10**	−0.16***	-					
12 Friends support	−0.01	0.05	0.25***	0.20***	0.20***	0.21***	0.22***	−0.16***	−0.09**	−0.09**	0.48***	-				
13 Significant others support	0.07*	0.05	0.29***	0.23***	0.22***	0.25***	0.26***	−0.17***	−0.08*	−0.11**	0.63***	0.75***	-			
14 Internet addiction factor1	−0.17***	−0.03	−0.12***	−0.07*	−0.09**	−0.03	−0.13***	0.20***	0.15***	0.22***	−0.16***	−0.05	−0.12***	-		
15 Internet addiction factor2	−0.06	0.03	−0.20***	−0.05	−0.09**	−0.10**	−0.14***	0.28***	0.21***	0.26***	−0.21***	−0.13***	−0.14***	0.64***	-	
16 Internet addiction factor3	−0.10**	−0.05	−0.165**	−0.03	−0.06*	−0.07*	−0.11**	0.27***	0.18***	0.25***	−0.22***	−0.10**	−0.15***	0.73***	0.76***	-
M	-	15.41	4.97	4.87	5.44	4.28	4.91	2.91	2.98	3.3	4.96	4.99	4.97	2.71	2.96	2.69
SD	-	1.42	1.03	1.29	1.26	1.35	1.17	0.89	0.87	0.94	1.37	1.24	1.26	1.13	1.27	1.11

*Gender was dummy coded such that 0 = female and 1 = male; Internet addiction factor1-3 were derived from item parceling*.

* *p < 0.05*,

** *p < 0.01*,

*** *p < 0.001*.

### Testing for the Measurement Model

In the measurement model, future expectations, PSS, and IU were treated as latent variables. Meanwhile, given that the internet addiction questionnaire is a one-dimensional scale, the factorial algorithm's balanced method was adopted to package 10 items into three factors (40). Internet addiction was also treated as a latent variable. In this way, the measurement model with four latent variables and 14 observation variables was finally obtained. The measurement model parameters were tested and estimated using the maximum likelihood method of the covariance structure model. Missing data were handled using the full maximum-likelihood estimation procedure. Overall, the measurement model fit well with the data (χ^2^/*df* = 4.55, CFI = 0.94, TLI = 0.93, RMSEA = 0.064, 90% CI [0.057, 0.071], SRMR = 0.045). As shown in Table 2, the latent variables' standardized factor loadings were significant (*p* < 0.001). The correlation coefficient of each latent variable is shown in Table 3. Specifically, future expectations were negatively correlated with IU and internet addiction and positively correlated with PSS. IU was negatively correlated with PSS and positively correlated with internet addiction, while PSS was negatively correlated with internet addiction.

**Table 2:** Measurement model: latent variable factor loadings.

	**Unstandardized factor loading**	**Standard error**	***t***	**Standardized factor loading**
**Future expectations**				
Work and education	0.73	0.04	18.05	0.67***
Marriage and family	0.55	0.05	11.14	0.43***
Children's future	0.70	0.05	13.75	0.53***
Social group	0.81	0.05	16.34	0.60***
Health	0.79	0.05	15.84	0.60***
**Intolerance of uncertainty**				
Prospective anxiety	0.88	0.04	25.41	0.77***
Prospective behavior	1.05	0.04	27.32	0.82***
Inhibitory behavior	0.95	0.03	29.51	0.87***
**Perceived social support**				
Family support	0.88	0.04	20.43	0.65***
Friends support	0.97	0.04	25.42	0.77***
Significant others support	1.22	0.04	33.14	0.96***
**Internet addiction**				
Internet addiction factor1	0.75	0.03	22.47	0.76***
Internet addiction factor2	0.84	0.03	24.58	0.83***
Internet addiction factor3	0.68	0.04	17.07	0.59***

*** *p < 0.001*.

**Table 3:** Correlation coefficients of latent variables.

	**Future expectations**	**Intolerance of uncertainty**	**Perceived social support**	**Internet addiction**
Future expectations	-			
Intolerance of uncertainty	−0.27***	-		
Perceived social support	0.43***	−0.19***	-	
Internet addiction	−0.31***	0.40***	−0.12**	-

** *p < 0.01*,

*** *p < 0.001*.

### Testing for Mediation

**Figure 2:**
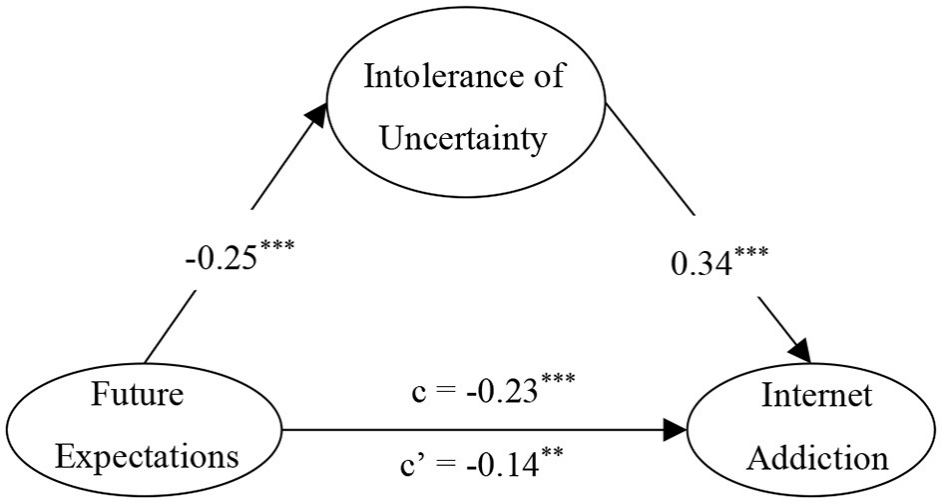
The mediating effect of intolerance of uncertainty in the association between future expectations and internet addiction. **p < 0.01, ***p < 0.001.

All covariates were held constant during the analysis. First, the direct effect of future expectations on internet addiction was tested, and the results showed that the model fit the data well (χ^2^/*df* =3.36, CFI = 0.99, TLI = 0.98, RMSEA = 0.048, 90%CI [0.036, 0.062], SRMR = 0.027). Future expectations significantly predicted internet addiction (γ = −0.23, 95% CI [−0.32, −0.14], *p* < 0.001), and explained 5.9% of the total variance in internet addiction. Next, IU was added as a mediator variable based on the original model. As shown in Figure 2, the results also showed that the model was a good fit with the data (χ^2^/*df* = 2.50, CFI = 0.99, TLI = 0.98, RMSEA = 0.039, 90% CI [0.028, 0.049], SRMR = 0.026. Future expectations significantly predicted IU (γ = −0.25, 95% CI [−0.33, −0.18], *p* < 0.001); IU significantly predicted internet addiction (γ = 0.34, 95% CI [0.25, 0.42], *p* < 0.001). Future expectations still significantly predicted internet addiction (γ = −0.14, 95% CI [−0.23, −0.06], *p* < 0.01), but the effect was weakened. Future expectations and IU explained 13.9% of the total variance in internet addiction. The results of the analyses suggested that IU had a significant mediating effect between future expectations and internet addiction. The mediating effect size was −0.09, 95% CI [−0.12, −0.06], *p* < 0.001, and the mediation effect accounted for 37.0% of the total effect.

### Testing for Moderated Mediation

**Figure 3:**
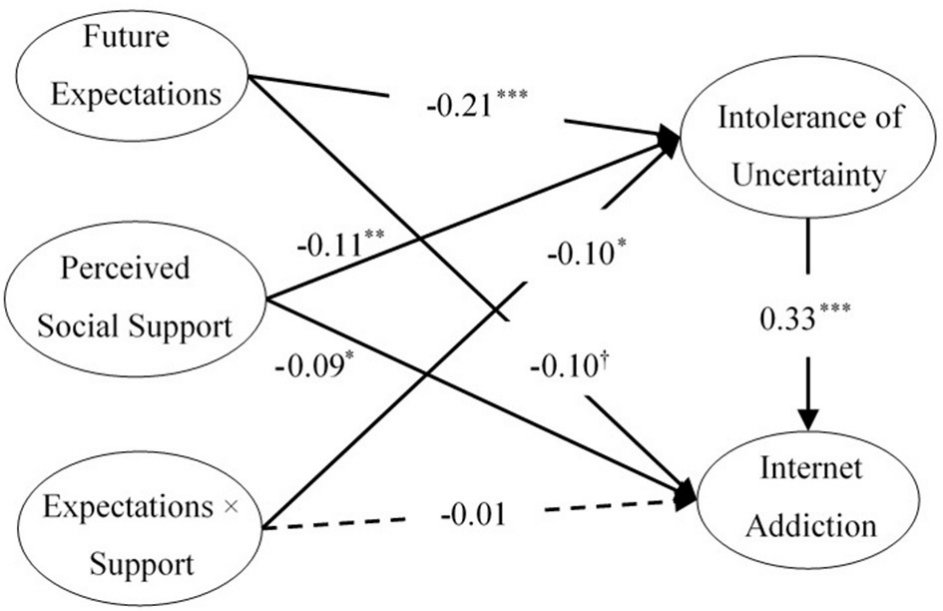
The moderating effect of perceived social support. The dotted line indicates that the coefficients are not significant. †*p* < 0.10, *p < 0.05, **p < 0.01, ***p < 0.001.

The latent moderated structural equation was applied to examine the moderating role of PSS (41). Two models were established in this study. In model 1, the main effects of PSS were added based on the mediation model of IU. The results showed that Model 1 fit well with the data (χ^2^/*df* = 2.98, CFI = 0.98, TLI = 0.97, RMSEA = 0.044, 90% CI [0.037, 0.052], SRMR = 0.037). The model explained 14.5% of the variance in internet addiction. As seen in Figure 3, in model 2, the latent interaction variable (future expectation × perceived social support) was added based on model 1. The researchers tried to assess whether model 2 was better than model 1. The model fit was assessed using the likelihood ratio to do this. In model 1 without the latent interaction variable (LogL [restricted model] = −17641.642) and in model 2 with the latent interaction variable (LogL [full model] = −17638.564), LR (*df* = 2) = 6.156, *p* < 0.05. These results indicate that model 2 trumped model 1. As shown in Figure 3, PSS significantly moderated the relationship between future expectations and IU (γ = −0.10, *p* < 0.05), but it did not moderate the association between future expectation and internet addiction (γ = −0.01, *p* > 0.05). Thus, PSS played a moderating role in the first half of the mediating effect.

**Figure 4:**
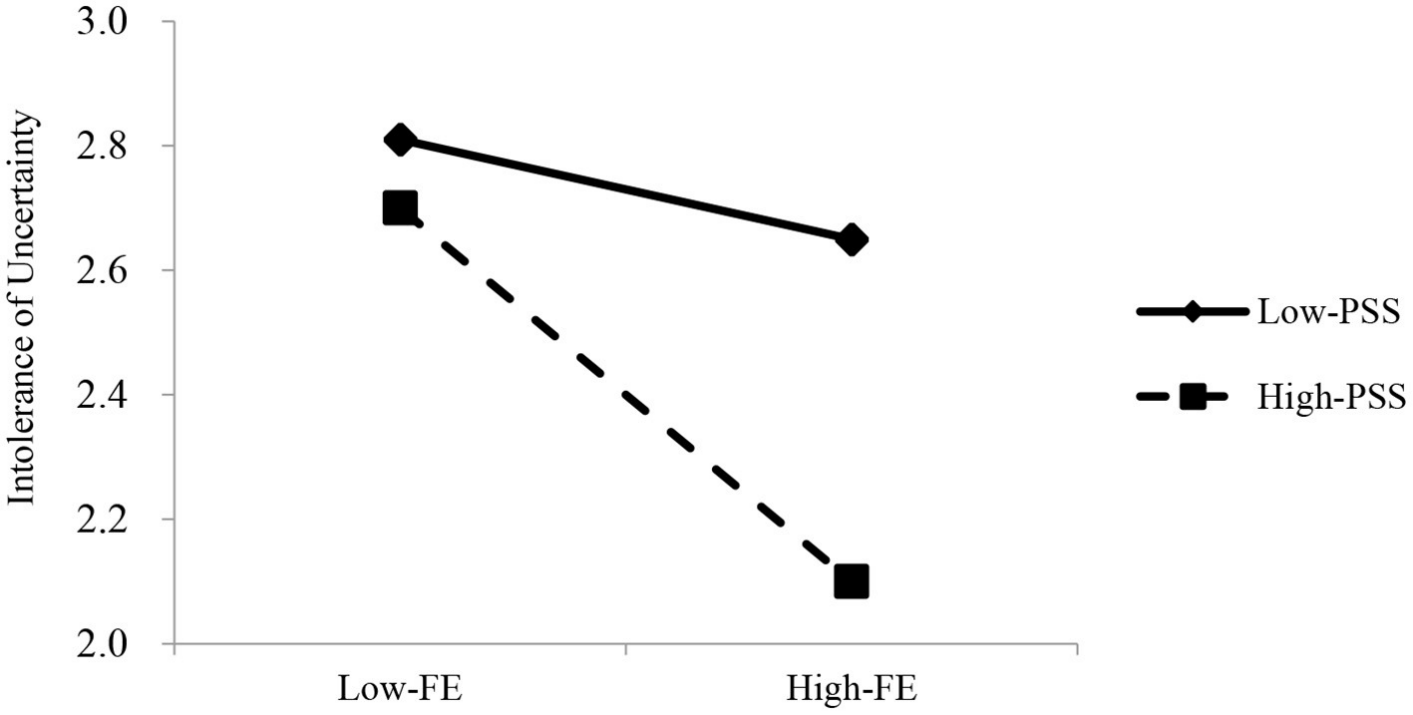
The moderating effect of perceived social support between future expectations and intolerance of uncertainty.

To further probe the interaction effect, a simple slope analysis was conducted. Participants were analyzed at two levels (low level, M—SD; high level, M + SD) according to the moderator's levels. Figure 4 shows that higher levels of future expectations (1 *SD* above the mean) were more strongly associated with IU when adolescents reported higher levels of PSS (1 *SD* above the mean) (β = −0.15, SE = 0.04, *t* = −3.61, *p* < 0.001). However, for adolescents with lower levels of PSS (1 SD below the mean), the effect of future expectations on IU was not significant (β = −0.04, SE = 0.04, *t* = −1.10, *p* > 0.05). That is, PSS moderated the indirect association between future expectations and internet addiction through IU.

## Discussion

Despite burgeoning evidence for the role of future time orientation in reducing internet addiction in adolescents, little is known about the mediating and moderating mechanisms underlying this relationship. To address this gap, this study established a complex theoretical model that explored both mediating (i.e., IU) and moderating (i.e., PSS) mechanisms in the association between future expectations and internet addiction among Chinese adolescents.

### The Mediating Role of Intolerance of Uncertainty

This study showed that future expectations significantly and negatively predicted internet addiction. This supports our hypothesis and is consistent with previous studies on the negative association between future time perspective and internet addiction [e.g., (15, 16)]. It further suggests drawing attention to future thinking when preventing and intervening internet addiction in adolescents. The existing literature has pointed out that the future time perspective is a cognitive-motivational concept (42). Besides playing a cognitive role in human functioning, future time perspective has a powerful motivational function. The motivational function for future expectations, the “prospective life course” perspective of future time orientation, enables individuals to maintain their stated goals and remain undistracted so that dependence on the internet in terms of time and energy is significantly discounted. It is also in line with the expectancy value theory (13), which assumes that the expectations of success probability and relative value are the key determinants of behavioral choices. If adolescents believe that future events are more likely to happen (positive expectations), they would prefer to focus on actions that help them achieve expectations. Meanwhile, one crucial reason teenagers are addicted to the internet is that they cannot meet their daily needs, thereby turning to the online world for comfort—typically, online games, dating, and chatting (43). If there is constructive compensation in real life, teenagers will not seek satisfaction online. For example, when individuals have higher goals and expectations, they will know where to go forward and pursue what satisfies them so as to avoid overuse of the internet.

The findings showed that future expectations significantly and negatively predicted IU. In the face of environmental uncertainty, individuals who have intrinsic motivations and positive qualities would tolerate uncertainty to a certain extent and be more consistent with common goals. Vela et al., (44) found that hope and search for meaning can significantly and positively predict grit, characterized by persistence in goals in uncertain situations. Although there is uncertainty in the future, uncertainty also comes with many possibilities. These possibilities reflect the nature of humans' expectations and probably make people believe that positive future events will happen. How do we develop specific coping mechanisms in the face of numerous uncertainties? This study provides an impressive perspective, namely, cultivating the capability to think about the future. Believing that positive future events are more likely to happen brings confidence and direction to adolescents, reducing personal uncertainty. Therefore, it would be said that future expectations display a compensating effect on IU.

IU significantly positively predicted internet addiction. The occurrence of internet addiction is inseparable from individual cognitive factors. As a cognitive bias, IU usually leads to many adverse outcomes (e.g., negative emotions and impulsive behaviors). Previous studies have shown that IU is a risk factor for social phobia, obsessive-compulsive disorder, generalized anxiety disorder, and symptoms of depression (45). This study's findings align with those of (46), revealing that community adults with high fear of the unknown (high IU) may experience cyberchondria and excessive use of the internet. These findings provide more evidence for the prevention and intervention of internet addiction. IU may be a trait variable and a state variable (47), so it is possible to provide useful guidance for individuals with high IU. For example, relying on families and teachers' cooperation, adolescents with high IU could change their thinking styles from “negative problem orientation” to “positive problem orientation.” They must accept that solving these problems requires more time and effort, and they believe that they can rely on their abilities to deal with issues rather than go online for hedonistic activities. From the perspective of clinical application, cognitive behavioral therapy provides cognitive reconstruction strategies for this maladaptive cognition to achieve a rational use of network functions (48).

Consistent with this hypothesis, the study indicates that IU mediated the relationship between future expectations and internet addiction. This suggests that apart from the direct effect of future expectations on internet addiction, IU may play an essential role in this relationship. Adolescence, a transition period from childhood to adulthood, is a critical period for individuals' development and usually involves various simulations and temptations in their environment. Although the internet does bring convenience to adolescents, its freshness, anonymity, and excitability may breed addiction. It appears that, when challenged with problems or behavioral concerns, serving as a protective factor (49), positive future expectations are conducive to making individuals tolerate some uncertainty (high tolerance of uncertainty) and focus on the task. This may reduce or even eliminate internet addiction behavior.

### The Moderating Role of Perceived Social Support

This study showed that PSS only moderates the link between future expectations and IU (the first half of the mediating link). As expected, the relationship between future expectations and IU was significant for adolescents with a high PSS level and not substantial for adolescents with a low PSS level. These findings indicate that adolescents with a high PSS level may develop an adaptive cognitive style, such as tolerance of uncertainty if they have a high level of future expectations. However, adolescents with a low level of PSS in their real life are not likely to choose an adaptive cognitive style, even if they have a high level of future expectations. How can we reduce individual IU? As mentioned above, it is necessary to focus on cultivating individuals' abilities of future thinking and improving the degree of PSS. As the stress-buffering model (25) emphasizes, PSS may buffer individuals from the influence of adverse situations. Social support is inherently characterized by intimacy and assistance. Therefore, the moderating role of PSSs could be understood in terms of individuals using external resources to enhance the positive impact of future expectations on IU to reduce personal uncertainty.

However, the study did not find that PSS moderated the relationship between future expectations and internet addiction (the direct pathway). This result could be explained by the potent role that future expectations play in adolescent lives. We argue that expectations are fundamental and too pervasive human motivation, which profoundly impacts adolescent emotions, cognitions, and behaviors. Therefore, individuals with high future expectations should possess low-intensity internet addiction, regardless of whether they have a high or low PSS level. The findings also demonstrate that the influence of individuals' internal traits on their behaviors is gradually stable in the adolescent period. In this study, however, both future expectations and PSS had independent effects on internet addiction. Further, PSS was negatively associated with internet addiction, in line with prior research, revealing that cumulative risk is related to adolescent internet addiction (50). If adolescents' social support is insufficient or there are not enough perceived resources in their environment, they may seek satisfaction in other areas such as the virtual world.

### Limitations and Future Directions

Despite the novelty of our data and its essential literary contribution, it is subject to some limitations. First, we employed a cross-sectional design that made it difficult to infer causality. Future researchers should use longitudinal designs to confirm causal hypotheses. Specifically, previous studies have obtained age differences in individuals' future orientation and delay discounting (51). Therefore, future research could explore the trajectory of future expectations and their longitudinal associations with internet addiction. Second, future expectations were mainly about positive future expectations and less about negative future expectations (or future fear, future anxiety). If both positive and negative future expectations were measured, the results would be more reliable and generalizable. Additionally, the measures were based on self-reports; future studies should try to collect data from multiple informants (e.g., parents' expectations and teachers' expectations) to strengthen the conclusions further. Third, although the Future Expectations Scale for Adolescents has been translated into Chinese, it is still derived from Western culture. Culture shapes the way individuals think about the past, present, and future time. Existing research has shown that Chinese teenagers pay more attention to their parents' happiness and health regarding hopes and fears for the future (52). However, this attentiveness has not been reflected in measurement tools. Fourth, additional research is needed to design to capture specific internet use (e.g., social media platforms, video games, shopping, surfing, etc) which would be timely and far more useful.

### Implications

Despite these limitations, our findings may contribute to understanding internet addiction and its potential protective and risk factors. First, the present study supports early motivation theories that emphasize the crucial role of expectations in behavior regulation (13). Our findings support the notion that adolescents' future expectations decrease internet addiction risk, highlighting the need to implement future thinking cultivation programs. Second, our study provided additional evidence that IU may be a mechanism linking future expectations and internet addiction, suggesting that interventions that target Internet addicts should help them establish more reasonable belief systems that allow them to cope with the uncertain experience. Third, the study indicates that perceived social support may help protect adolescents from low IU levels associated with high levels of future expectations. This shows that one crucial component in intervention programs is enhancing students' social support networks (30) and assisting the youth in developing the necessary skills to access those networks.

## Conclusion

The evidence from our study suggests that future expectations are protective factors for internet addiction in adolescents, and this effect is partially mediated by IU. Additionally, PSS plays a moderating role in the association between future expectations and IU in adolescents. These findings suggest that adolescents' future expectations, IU, and PSS may be key targets for prevention and intervention programs dealing with internet addiction. This study will help to inspire future researchers to better understand the phenomenon of internet addiction and advance the field of internet research.

## Data Availability Statement

The raw data supporting the conclusions of this article will be made available by the authors, without undue reservation.

## Ethics Statement

This study was approved by the ethics committee of Faculty of Psychology at Southwest university. Written informed consent to participate in this study was provided by the participants' legal guardian/next of kin.

## Author Contributions

GD and HL conceived and designed experiments. GD contributed to the data analysis and writing. HL revised it critically for important content. All authors contributed to the article and approved the submitted version.

## Conflict of Interest

The authors declare that the research was conducted in the absence of any commercial or financial relationships that could be construed as a potential conflict of interest.

## Publisher's Note

All claims expressed in this article are solely those of the authors and do not necessarily represent those of their affiliated organizations, or those of the publisher, the editors and the reviewers. Any product that may be evaluated in this article, or claim that may be made by its manufacturer, is not guaranteed or endorsed by the publisher.
